# Functional Stroke Mimics: Patient Characteristics, CT‐Based Multimodal Imaging and Long‐Term Outcome in a Comparative Cohort Study

**DOI:** 10.1111/ene.70617

**Published:** 2026-05-06

**Authors:** Filipa Bastos, Davide Strambo, Alexander Salerno, Vincent Dunet, Selma Aybek, Patrik Michel

**Affiliations:** ^1^ Stroke Centre, Service of Neurology, Department of Clinical Neurosciences Lausanne University Hospital and University of Lausanne Lausanne Switzerland; ^2^ Neuroradiological Unit, Service of Diagnostic and Interventional Radiology, Department of Medical Radiology Lausanne University Hospital and University of Lausanne Lausanne Switzerland; ^3^ Neurology, Faculty of Science and Medicine Fribourg University Fribourg Switzerland

**Keywords:** CT, functional neurological disorder (FND), functional stroke mimic (FSM), imaging, long‐term‐outcome, outcome

## Abstract

**Background and Purpose:**

Functional stroke‐like episodes (FSMs) are an increasingly recognised stroke mimic with demographic and clinical characteristics that differ from acute ischaemic strokes (AISs) but have unclear long‐term outcomes.

**Materials and Methods:**

We report retrospective data on consecutive patients with FSM who underwent acute perfusion‐CT (PCT) admitted to Lausanne University Hospital (2003–2017). We compared them to all contemporaneous AISs undergoing PCT from the Acute‐STroke‐Registry‐and‐Analysis‐of‐Lausanne (ASTRAL).

**Results:**

Twenty‐five FSMs and 3201 control‐AISs were included. FSM patients were significantly younger (median 43 vs. 73 years, adjusted odds ratio (OR_adj_) 0.92), had a higher incidence of psychiatric disorders (OR_adj_ 5.33/17.07), and over half had a prior history of neurological and non‐neurological functional disorders. FSM patients more often presented decreased vigilance (OR_adj_ = 9.28) and sensory deficits (OR_adj_ = 3.87), and less visual field defects (OR_adj_ = 0.14) and dysarthria (OR_adj_ = 0.20). FSM patients showed no significant changes on plain‐CT and PCT. Acute revascularisation rates were similar in both groups (48% vs. 43%). Follow‐up at 3‐months revealed significant handicap in 41% of patients, similar to the control group in propensity‐score‐matched analysis, and lower mortality (0% vs. 20%, *p*_adj_ 0.04). After a median of 9 years follow‐up, FSM patients failed to functionally improve further and 55% experienced additional functional neurological events.

**Conclusion:**

In this single‐centre cohort of consecutive FSMs undergoing acute PCT, we identified distinctive demographic and clinical features, normal CT‐based neuroimaging, but still a high thrombolysis rate. Long‐term observation revealed a high rate of recurrent functional events and persistent disability, suggesting the need for more effective treatment and regular follow‐up.

## Graphical abstract

Functional stroke‐like episodes are increasingly recognised as stroke mimics. In a study of 25 cases who underwent hyperacute multimodal CT, patients were younger than their counterparts with acute ischaemic strokes and often had a psychiatric and functional disorder background. Despite normal imaging and inconsistent presentation, revascularisation treatment rates were similar to true strokes. Long‐term follow‐up (median 9 years) showed persistent disability and frequent recurrence, highlighting the need for improved early recognition and management.

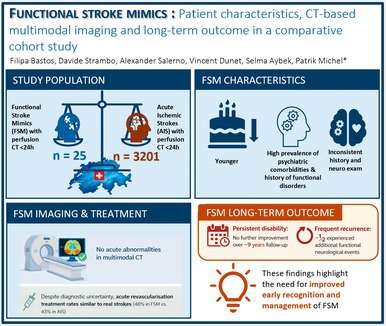

## 1 Introduction

Functional neurological disorder (FND) is frequent and can mimic nearly every type of neurological condition [1, 2]. Among these, functional episodes mistaken for strokes are an increasingly recognised stroke mimic, with reported incidence ranging from 10% to 15% [3, 4] to up to 30% of all stroke mimics [5], and accounting for 2% of all suspected strokes [3, 4]. Functional stroke mimics (FSMs) are particularly frequent in the acute setting, especially among patients receiving intravenous thrombolysis (IVT) [6, 7], as the focus is on minimising treatment delay, which may result in less precise assessments, increasing the risk of misdiagnosing clinical presentations that resemble stroke. On the other hand, strokes can sometimes present with functional‐like symptoms, a phenomenon known as ‘stroke chameleons’ [8].

A metanalysis suggested that FSM patients have distinct characteristics compared to other stroke mimics and acute ischaemic strokes, sharing similarities with other FNDs. They are younger, more frequently female and present with more weakness and numbness, but less language and consciousness impairment than stroke patients and medical mimics [3]. Additionally, they often have a high rate of comorbid psychiatric disorders although this has not been consistently identified [3, 9]. In a recent case series of 14 FND patients following cerebrovascular disease, most had functional symptoms localised to cerebrovascular lesions; interestingly these lesions mapped to regions identified as disrupted networks in FND patients [10].

Although newer classification systems of FND in general, including FSM, emphasise a proactive phenotype‐based diagnosis over the simple exclusion of other neurological disorders, data on specific functional signs at presentation of FSM remain scarce [6, 11, 12]. A prospective, multicentre, observational study of 56 patients, including both stroke and non‐stroke cases with functional neurological symptoms (40 presenting with isolated functional manifestations), identified positive functional signs, anatomical inconsistencies, and atypical presentation in approximately one quarter of cases [13]. A retrospective analysis of a small Italian cohort found a positive Hoover sign in 10 of the 24 FSM patients [5]. While an atypical clinical presentation, alongside the absence of acute or subacute vascular anomalies on neuroimaging, may support a functional origin, it does not definitively establish the diagnosis [6].

FSM IVT rates vary considerably between studies ranging from 3% to 31% depending on the study population and setting (Gargalas et al. 2015, London, *n* = 98: 3% [14]; Wilkins et al. 2017, Middle East, *n* = 161: 10% [15]; Jones et al. 2020, *n* = 40: 13% [13]; Caruso et al. 2024, Italy, *n* = 84: 31% [16]).

The prognosis of FSM remains unclear, with conflicting findings. Some studies report poor outcomes overall [13, 17], whereas others show better outcomes than in other motor FNDs [2, 6, 14, 18, 19]. A prospective cohort by Jones et al. suggests that recovery can be slow, but their cohort did not only include FSM [13]. A small Dutch study of 38 FSM patients revealed that at a median of 28 months follow‐up, self‐rated outcomes were worse than those of patients with mild acute ischaemic stroke (AIS) treated with IVT [17]. Caruso et al. reported 37% recurrence at 3 months in their 84 FSM patients, but without further detail [16]. Long‐term recurrence rate and type in FSM patients remain largely unknown.

The objective of this study, assessing quality of care in our institution, was to identify FSM patient characteristics and comorbidities compared to AIS patients, describe the acute cerebral perfusion CT imaging and present long‐term functional outcome and recurrence data. Based on the available literature and our clinical and theoretical background, we hypothesised that FSM patients would be younger, more often female, have similar clinical deficits but inconsistent findings in history taking and physical examination. We did not anticipate finding any relevant imaging abnormalities and we speculated that IVT rates would be significantly lower than in AIS. Although long‐term outcome data are scarcely available for FSM patients, we expected these patients to have a lesser long‐term handicap than AIS patients.

## 2 Materials and Methods

### 2.1 Patients and Case Definition

We prospectively identified consecutive patients over 18 years old (no upper age limit) who were admitted to the Stroke Unit of the Lausanne University Hospital between 2003 and 2017 with an initial suspicion of acute ischaemic stroke (AIS), who underwent acute perfusion CT (PCT) within 24 h of symptoms onset or of last known well (if unknown onset), and were ultimately diagnosed with an FSM.

The case definition of FSM required an initial clinical suspicion of AIS based on an acute onset of neurological deficit potentially compatible with a focal cerebral dysfunction with no evidence of acute ischaemic lesions on acute or follow‐up brain parenchymal imaging (MRI when available, otherwise CT), and a subsequent clinical course inconsistent with AIS, for example, the neurological status during hospitalisation was ultimately incompatible with a diagnosis of stroke. Positive functional signs were sought [6], but their presence was not part of our case definition as they are not pathognomonic of FSM and can also be found in AIS with functional overlay [13]. The final diagnosis of FSM was made by experienced board‐certified neurologists based on thorough history taking, clinical course, physical examination, imaging and psychiatric evaluation when deemed needed. Differential diagnosis with other stroke mimics was always considered and then excluded based on all the available clinical, imaging and laboratory data. Transient Ischemic Attacks (TIA), that is, stroke‐like symptoms lasting less than 24 h, were not included in the study; therefore, the symptoms of all our FSM patients lasted by definition more than 24 h. Patients with presumed functional symptoms but with uncertainty about the final diagnosis, such as functional overlay [13], were excluded. These rather restrictive inclusion criteria were chosen in order to select ‘pure’ FSM patients and avoid ‘noise’ from comparing imprecisely defined populations.

As control group we selected all consecutive AISs admitted during the same period from the Acute‐Stroke‐Registry‐and‐Analysis‐of‐Lausanne (ASTRAL) who underwent PCT within 24 h. ASTRAL is a single centre cohort study of all adult (no upper age limit) AIS patients since 2003 arriving within 24 h of last‐well time at our hospital and being admitted to the stroke unit and/or intensive care unit [20].

### 2.2 Data Collection

Electronic medical records were reviewed for demographics (age, biological sex, ethnicity), prestroke handicap (modified Rankin score, mRS), prehospital metrics (type of onset, onset‐to‐hospital arrival time), cardiovascular risk factors (CVRFs) (hypertension, diabetes mellitus, hypercholesterolaemia, cardiac and peripheral arterial disease, smoking, alcohol), physical and neurological comorbidities (active cancer, migraine), psychiatric history and treatment and history of neurological and non‐neurological functional disorders. Psychiatric comorbidities were divided into two categories (‘depression‐related disorders’ and ‘psychosis‐related disorders’, as defined in the Elixhauser comorbidity index that includes a list of precisely defined psychiatric conditions based on ICD‐9 codes) [21]. We chose to do so to allow comparability between the two study populations, given this is the variable recorded in our stroke registry (ASTRAL). Clinical deficits on arrival were recorded according to their presence and definition in the National Institutes of Health Stroke Scale (NIHSS). This includes the term ‘vigilance’ which is assessed in the NIHSS on a descriptive scale of normal–somnolence–stupor–coma. In patients with FSM we reviewed the medical records for the presence of functional positive signs at the baseline neurological assessment, such as give‐away weakness, drift without pronation, Hoover sign, global pattern of weakness, or unilateral facial lip pulling [6].

### 2.3 Neuroradiology

Multimodal CT was the standard of care for suspected AIS in our emergency department at the time of this study [22]. Acquisition included non‐contrast (NCCT) series, CT angiography (CTA), PCT and post‐contrast series. All CT parameters are reported in the Supporting Information. Iodinated contrast injection for CTA and PCT was performed as standard of care, except in patients with specific contraindications including creatinine clearance below 30 mL/min/1.73 m^2^ (50 if patient was under metformin) or known contrast allergy as previously published [15]. PCT was performed using two intravenous bolus injections of contrast and a multidetector CT in cine mode.

An experienced diagnostic neuroradiologist (VD) unaware of the diagnosis or the clinical presentation other than ‘suspicion of stroke’ reviewed all FSM patient CT images. The NCCT was analysed for the presence of leukoaraiosis, chronic stroke lesions and early ischaemic changes. CTA source images and maximum‐intensity projections were analysed for any signs of atherosclerosis and for focal arterial stenosis ≥ 50% or occlusion. PCT data were analysed using the central volume principle to create parametric maps of relative cerebral blood volume, mean transit time (MTT) and relative cerebral blood flow. Focal hypoperfusion (FHP) was defined as a clearly prolonged MTT [22] compared to the contralateral mirror area on visual assessment, visible on ≥ 2 consecutive slices and not attributable to an underlying chronic tissue lesion.

Acute imaging results from control AIS patients were used from ASTRAL, stemming from review of images by an experienced stroke physician, the neuroradiology report, and in cases of doubtful findings a joint discussion from weekly multidisciplinary meetings involving at least one senior neurologist and one senior neuroradiologist.

Repeat brain CT or MRI was performed if needed in the subacute phase to better assess ischaemic and haemorrhagic changes and stroke aetiology.

### 2.4 Outcomes

For all patients, outcome variables were disability measured by the modified Rankin Scale (mRS) at 3 and 12 months and recurrent cerebrovascular events within 12 months of the index event. These data were obtained from either in‐person visits at the routine clinical outpatient follow‐up or through a structured telephone interview by Rankin‐certified personnel. In FSM patients, we additionally collected data on FND recurrence. Long‐term outcome data for FSM patients were obtained from 2020 to 2022 in a telephone interview conducted by one of the authors (FB) with the patient (or next‐of‐kin if the patient was not available), covering the entire period from the index event (structure of the telephone interview in the Supporting Information). If the patient had visited emergency departments or had been hospitalised during this follow‐up, information was completed from medical records from our institution or other hospitals.

### 2.5 Statistical Analysis

We summarised continuous variables as median values with interquartile range and categorical variables as absolute numbers and percentages. We performed univariate screening of independent baseline and outcome variables comparing them between FSM and AIS‐control groups using Pearson's chi‐squared test for categorical variables and Mann–Whitney *U* tests for continuous variables, as appropriate.

To identify patient baseline features independently associated with FMS compared to AIS, we performed a multivariable regression analysis using a binary logistic regression model with the group variable (FSM vs. AIS) as the dependent variable. Covariates included in this model were baseline features associated with FSM in the univariable analysis at a significance level of *p* < 0.1. Variable selection in the regression model was then performed using stepwise backward elimination based on the Akaike Information criterion (AIC). A second multivariable logistic regression analysis with a similar structure was performed, including as covariates variables describing the presence or absence of each neurological deficit at baseline. Finally, to assess an outcome difference between AIS and FSM, we used an ordinal logistic regression model with the 3‐month mRS as dependent variable (‘Rankin‐shift’) and the group variable (AIS vs. FSM) as predictor. To adjust for baseline imbalances between groups, we used propensity score weighting and matching (PSM), with weights estimated via a binary logistic regression model including as covariates baseline patient features potentially associated with the outcome. Results of the multivariable analyses are reported as odds ratio (OR) with corresponding 95% confidence interval (CI). A *p*‐value < 0.05 was considered statistically significant. For statistical analysis, we used R statistical software (version 4.4.1, R Core Team [2016], R Foundation for Statistical Computing, Vienna, Austria).

### 2.6 Ethical Considerations

This study was conducted according to the legislation of the Canton de Vaud, Switzerland. The Ethics Commission for Research on Humans of the Canton de Vaud and the Medical Director of the institution have authorised the collection of prospective outcome data of FSM patients with informed consent of the patient (or next of kin if the patient was unavailable) (ethics protocol ID 2017‐00472) and granted the right to access medical records to coauthors not directly involved in the care of these patients. For patients where neither the patient nor next‐of‐kin were available, we only analysed baseline data after anonymisation, as authorised in the Swiss Human Research Act (HRA) for patients where there are no indications that they (or his/her legal representative) refused such an analysis. For the control group from the ASTRAL registry, there was no need for patient consent or for ethical commission approval according to the HRA (art. 3) and the applicable data protection legislation given that this was a quality assessment of our institution.

## 3 Results

Of 26 initially identified FSM patients who underwent acute PCT, one was excluded because of uncertainty in the final diagnosis (more likely post‐ictal limb paresis with functional overlay than pure FSM). In the remaining 25 patients, the official discharge diagnosis was FSM or probable FSM in 22/25 (88%), unexplained neurological symptoms in 2/25 (8%), and imaging‐negative lacunar stroke in 1/25 (4%). In this last patient, the diagnosis was changed to FSM 2 months later after readmission for a recurrent FSM event. In the study observation period, 3201 AISs with acute PCT within 24 h were admitted to our institution (Figure S). The baseline data of the analysed populations are shown in Table 1.

**TABLE 1:** Univariate screening of independent variables in the overall cohort, FSM patients and AIS patients.

Variable	Overall population included (*n* = 3226)	AIS (*n* = 3201)	FSM (*n* = 25)	*p* (univ)	Odds ratio (95% CI)
Demographics					
Age	73.1 (61.2–81.4)	73.2 (61.6–81.5)	43 (35.3–54.7)	< 0.01	0.92 (0.90–0.94)
Biological sex, F	1434/3225 (44.5%)	1423/3200 (44.5%)	11/25 (44%)	1	0.98 (0.44–2.17)
White ethnicity	3102/3226 (96.2%)	3081/3201 (96.2%)	21/25 (84%)	0.01	0.20 (0.07–0.60)
Cardiovascular risk factors					
Arterial hypertension	2275/3226 (70.5%)	2268/3201 (70.8%)	7/25 (28%)	< 0.01	0.16 (0.07–0.38)
Atrial fibrillation	394/1538 (25.6%)	393/1513 (26%)	1/25 (4%)	0.02	0.12 (0.02–0.88)
Coronary artery disease	229/1533 (14.9%)	228/1508 (15.1%)	1/25 (4%)	0.21	0.23 (0.03–1.74)
Diabetes	596/3224 (18.5%)	593/3199 (18.5%)	3/25 (12%)	0.56	0.60 (0.18–2.01)
Dyslipidaemia	2429/3220 (75.4%)	2418/3195 (75.7%)	11/25 (44%)	< 0.01	0.25 (0.11–0.56)
Cancer, active	149/3211 (4.6%)	149/3186 (4.7%)	0/25 (0%)	0.53	0.00
Migraine, history of	169/3203 (5.3%)	163/3178 (5.1%)	6/25 (24%)	< 0.01	5.84 (2.30–14.82)
Smoking	777/3204 (24.3%)	760/3179 (23.9%)	17/25 (68%)	< 0.01	6.76 (2.91–15.73)
Alcohol abuse, active	324/3214 (10.1%)	321/3189 (10.1%)	3/25 (12%)	1	1.22 (0.36–4.09)
Previous medical history					
Cerebral vascular events	801/3212 (24.9%)	799/3187 (25.1%)	2/25 (8%)	0.08	0.26 (0.06–1.10)
Psychosis‐related disorders	273/3203 (8.5%)	265/3178 (8.3%)	8/25 (32%)	< 0.01	5.17 (2.21–12.1)
Depression‐related disorders	167/3215 (5.2%)	160/3190 (5%)	7/25 (28%)	< 0.01	7.36 (3.03–17.89)
Functional disorders	13/25 (52%)	Not available	13/25 (52%)	NA	NA
Onset				0.21	
Wake, known	2332/3226 (72.3%)	2310/3201 (72.2%)	22/25 (88%)	0.12	Ref.
During sleep	669/3226 (20.7%)	667/3201 (20.8%)	2/25 (8%)	0.18	0.31 (0.07–1.34)
Unwitnessed	225/3226 (7%)	224/3201 (7%)	1/25 (4%)	0.85	0.47 (0.06–3.49)
Metrics					
Onset‐to‐door (min)	158 (80–397)	158 (80–399.3)	115 (90.7–202)	< 0.01	1.00 (1.00–1.00)
mRS, prestroke	0 (0–1)	0 (0–1)	0 (0–0.3)	< 0.01	0.55 (0.30–0.99)
NIHSS, baseline	7 (4–15)	7 (4–15)	5 (4–9.3)	< 0.01	0.93 (0.86–1.00)
NIHSS, 24 h	4 (2–11)	4 (2–11)	3 (0.9–5.1)	< 0.01	0.90 (0.81–1.00)
Radiological variables					
NCCT, acute anomalies	1196/3179 (37.6%)	1196/3154 (37.9%)	0/25 (0%)	< 0.01	0.00 (0.00‐Inf)
NCCT, chronic stroke	1018/3070 (33.2%)	1015/3045 (33.3%)	3/25 (12%)	0.04	0.27 (0.08–0.91)
NCCT, leukoaraiosis	961/3070 (31.3%)	958/3045 (31.5%)	3/25 (12%)	0.06	0.30 (0.09–0.99)
CTA, significant atherosclerosis	2076/3022 (68.7%)	2070/2997 (69.1%)	6/25 (24%)	< 0.01	0.14 (0.06–0.36)
PCT, focal hypo‐ and/or hyperperfusion	2358/3220 (73.2%)	2357/3195 (73.8%)	1/25 (4%)	< 0.01	0.01 (0.00–0.11)
Acute treatment				0.06	
No revascularisation	1836/3225 (56.9%)	1823/3200 (57%)	13/25 (52%)	0.77	Ref.
IVT	986/3225 (30.6%)	974/3200 (30.4%)	12/25 (48%)	0.09	1.73 (0.79–3.80)
EVT+/‐IVT	403/3225 (12.5%)	403/3200 (12.6%)	0/25 (0%)	0.11	0.00 (0.00‐Inf)
Hospitalisation					
Length, days	9 (6–13)	9 (6–13)	6 (3.3–8.7)	< 0.01	0.90 (0.82–0.98)
Orientation at discharge				0.01	
Home	1142/3220 (35.5%)	1125/3195 (35.2%)	17/25 (68%)	< 0.01	Ref.
Rehab.	1004/3220 (31.2%)	1000/3195 (31.3%)	4/25 (16%)	0.15	0.26 (0.09–0.79)
Institution/EMS/other acute/hospital	829/3220 (25.7%)	825/3195 (25.8%)	4/25 (16%)	0.37	0.32 (0.11–0.96)
In‐hospital death	245/3220 (7.6%)	245/3195 (7.7%)	0/25 (0%)	0.29	0.00
Outcome					
mRS, 3 months	2 (1–4) (*n* = 3177)	2 (1–4) (*n* = 3199)	1 (0–3) (*n* = 22)	< 0.01	0.67 (0.49–0.92)
mRS, 12 months	2 (1–4) (*n* = 2432)	2 (1–4) (*n* = 2454)	1 (0–1) (*n* = 22)	< 0.01	0.52 (0.35–0.77)
mRS, last contact for FSM	NA	NA	1 (0–1)	1 (0–1)	NA
Mortality 12 months	585/3009 (19.4%)	585/2987 (19.6%)	0/22 (0%)	0.04	0.00
Recurrence					
Cerebrovascular events 12 months	200/2917 (6.9%)	200/2895 (6.9%)	0/22 (0%)	0.39	0.00
Functional events at last contact					
Recurrent FSM	8/22 (36.4%)	NA	8/22 (36.4%)	NA	NA
Recurrent functional events non‐FMS	NA	NA	12/22 (54.5%)	NA	NA
Recurrent FSM + other	3/22 (13.6%)	NA	3/22 (13.6%)	NA	NA

*Note:* Continuous and ordinal variables are expressed as medians (with interquartile range, IQR), and categorical variables as absolute counts (with percentage), unless stated otherwise.

Abbreviations: AIS, acute ischaemic stroke; CT/A, computed tomography/angiography; EVT, endovascular thrombectomy; F, female; FSM, functional stroke mimic; IVT, intravenous thrombolysis; min, minutes; mRS, modified Rankin Scale; NA, not available or not applicable; NCCT, non‐contrast CT; NIHSS, National Institutes of Health Stroke Scale; PCT, perfusion computed tomography; Ref, reference for odds ratio calculation.

### 3.1 Patient Characteristics

The 25 FSM patients were significantly younger than AIS patients (median age 43 years vs. 73 years, *p* < 0.001), with a similar biological sex distribution (44% females in both groups), a higher proportion of non‐white individuals (16.0% vs. 3.8%, *p* = 0.01), a lower prevalence of CVRFs and previous cerebrovascular events, and a higher prevalence of comorbid psychosis or depression‐related disorders (Table 1). More than half of the FSM patients had a prior history of functional neurological and non‐neurological disorders (*n* = 13/25, 52%), although this information was often not available in the acute phase. Of the 25 FSM patients, 15 had a psychiatric evaluation during the acute index hospitalisation. This information was not available for the AIS population. Granular information on psychiatric and functional comorbidities of the FSM cohort is available on Table S2.

FSM patients arrived at the hospital more rapidly than AIS patients (median onset‐to‐door time: 115 vs. 158 min, *p* < 0.01); their baseline NIHSS was significantly lower (5 vs. 7 points, *p* < 0.01), and a tendency for shorter hospital stays was observed in the univariate analysis (6 vs. 9 days, *p* < 0.01). In the multivariable logistic regression analysis, younger age, current smoking, medical history of psychosis and/or depression‐related disorders, absence of previous cerebral vascular event, and lower NIHSS were independently associated with FSM (Table 2).

**TABLE 2:** Results of the two multivariable logistic regression models: Upper part with demographics, risk factors and admission data; lower part with neurological deficits on admission.

Variable	Odds ratio (95% CI)	OR *p*
Demographics, risk factors and admission data		
Age	0.92 (0.89–0.94)	< 0.001
White ethnicity	0.34 (0.10–1.15)	0.082
Smoking	**3.97 (1.53–10.28)**	**0.005**
Migraine, history of	**2.90 (0.99–8.52)**	**0.053**
Psychosis‐related disorders, history of	**5.33 (1.90–15.02)**	**0.002**
Depression‐related disorders, history of	**17.07 (5.69–51.19)**	**< 0.001**
Cerebral vascular events, history of	0.17 (0.04–0.79)	0.024
Onset‐to‐door time (60 min)	0.84 (0.77–0.96)	0.010
Baseline NIHSS	0.91 (0.84–0.98)	0.017
Neurological deficits on admission		
Dysarthria	0.20 (0.07–0.52)	0.001
Sensory	**3.87 (1.56–9.61)**	**0.004**
Visual field defects	0.14 (0.04–0.47)	0.001
Vigilance	**9.28 (3.59–23.99)**	**< 0.001**

*Note:* Variables positively associated with functional stroke mimics are in bold for clarity.

Abbreviation: NIHSS, National Institutes of Health Stroke Scale.

### 3.2 Neurological Deficits at Baseline Evaluation

At baseline evaluation, FSM patients more frequently presented with a decreased level of consciousness and sensory findings; they less often had dysarthria or visual field defects (Table S1). These associations remained significant in multivariable analysis (Table 2). Positive functional signs were observed in 40% of FSM patients (*n* = 9/25) and clinical localisation of the deficit to a vascular territory was not possible in 40%. Overall, 96% of FSM patients (*n* = 24/25) had either positive functional signs or neurological symptoms not attributable to a vascular territory.

### 3.3 Radiological Features

No FSM patient showed ischaemic changes on acute non‐contrast CT compared to 37.9% of AIS patients. FSM patients also had significantly fewer chronic infarcts (12% vs. 33%, *p* = 0.04 in univariate comparison), numerically less but statistically not significant leukoaraiosis (12% vs. 31.5%, *p* = 0.06), and less precerebral or intracranial atherosclerosis on CTA (24% vs. 69%, *p* < 0.01). Acute‐phase PCT was normal in all but one FSM patient, whereas focal hypoperfusion was present in 73.8% (*n* = 2357/3197) of AIS patients (*p* < 0.01). The single FSM patient with abnormal PCT presented clinically with a witnessed onset of waxing and waning left‐sided hemiparesis, preceded by vertigo, perspirations, anxiety, and no cognitive impairment or headache. NCCT and CTA were unremarkable, but PCT showed a slight right hemispheric benign oligaemia that did not meet the threshold for ischaemia (Figure S2). Follow‐up MRI was normal. During hospitalisation, this patient's clinical course was characterised by inconsistencies in the reported history, fluctuating neurological findings not consistent with an acute ischaemic stroke, and multiple somatic complaints. Psychiatric evaluation identified a concomitant recurrent depressive disorder. Despite the PCT abnormality, all other clinical and radiological findings supported a functional episode, which was therefore retained as the final diagnosis. No plausible explanation for the right hemispheric benign oligaemia was identified.

### 3.4 Acute Treatment

The revascularisation treatment rates were similar in both groups (48% vs. 43%); 48% of FSMs were treated by IVT vs. 30.4% of AISs (non‐significant difference), and an additional 12.6% of AISs underwent acute endovascular treatment (EVT) ± IVT. There were no haemorrhagic complications following IVT in the 12 FSM patients.

### 3.5 Clinical Outcomes

**FIGURE 1:**
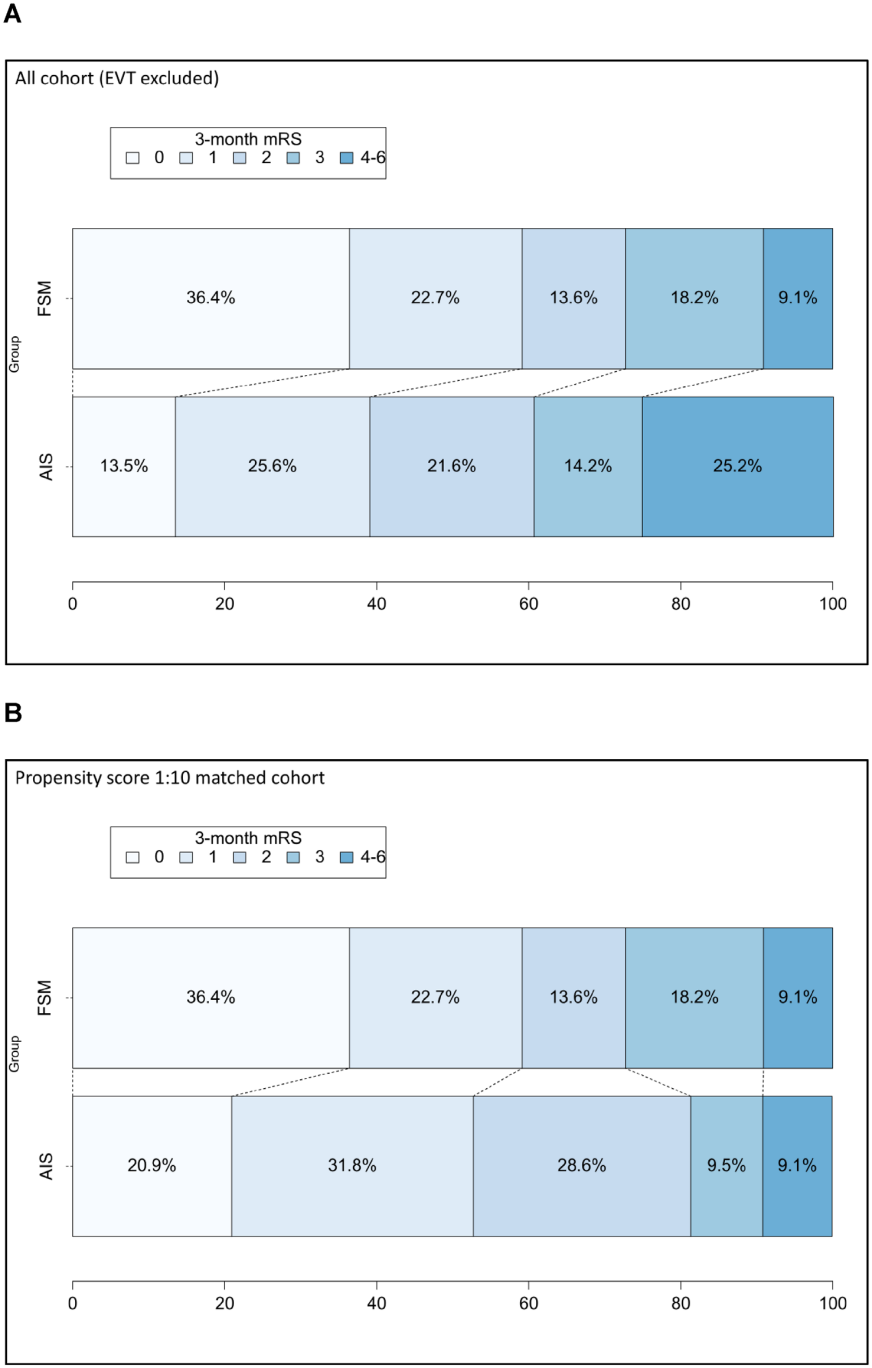
(A) Distribution of the adjusted modified Rankin scores (functional outcome) at 3 months; (B) Same, but using propensity‐matched patients only (matching for biological sex, history depression/psychosis, coronary artery disease, active cancer, NIHSS on admission, previous mRS, thrombolysis). AIS, acute ischaemic stroke; EVT, endovascular thrombectomy; FSM, functional stroke mimic; mRS, modified Rankin Scale; NIHSS, National Institutes of Health Stroke Scale.

Data on the functional outcome (mRS) at 3 and 12 months were available for 22 of 25 FSM patients and for 3199 and 2454 of 3201 AIS patients at 3 and 12 months, respectively. Median 3‐ and 12‐month mRS scores were significantly lower in FSM patients compared to AIS patients. 12‐month mortality was also lower in the FSM group. However, after PSM, the difference in the 3‐month mRS was no longer significant: psm‐cOR = 1.38 (95% CI 0.59–3.23, *p* = 0.45; variables used for matching: biological sex, history of depression/psychosis‐related disorders, coronary artery disease, active cancer, NIHSS on admission, previous mRS, IVT). The 3‐month mRS distribution in FSM patients, the AIS cohort and the ps‐matched AIS cohort are displayed in Figure 1A,B.

Extended follow‐up beyond 12 months was available for 22 of 25 FSM patients, with a median follow‐up of 9 years (range: 3–17 years). No further improvement in mRS was observed after the 12‐month follow‐up (Table 1). At the last available follow‐up, over half of the FSM patients (*n* = 12/22) had experienced at least one additional FND episode: 8 had recurrent FSMs, 3 experienced FSMs and other types of FNDs, and 1 developed only a non‐FSM FND (Table 1).

## 4 Discussion

In a single‐centre study of FSM patients undergoing multimodal CT, we found that these individuals were younger, more frequently of non‐white ethnicity, had a higher prevalence of psychiatric comorbidity, and presented with slightly less severe neurological symptoms compared to contemporary consecutive AIS patients. Acute CT‐based neuroimaging, including PCT, was essentially normal in FSMs, and the rate of IVT administration was high and similar to the control AIS group. Despite a better medium‐term disability outcome in FSM patients mainly driven by baseline differences (as shown by psm‐analysis), we observed that long‐term handicap and recurrent functional symptoms were common in this population.

As expected and reported in other studies of functional neurological disorders, our FSM patients were significantly younger than AIS patients. Unlike previous studies [3, 13, 16], we did not observe a female predominance. This discrepancy may reflect a selection bias in our cohort; a functional origin of symptoms might be more often suspected in female patients in an emergency evaluation, potentially leading to a decision not to perform multimodal neuroimaging and therefore exclusion from our case definition. We observed an over‐representation of non‐white ethnicity among FSM patients, a finding consistent with a previous study conducted in the Middle East [15]. One explanation for this could be that cultural factors may influence the expression of psychological distress through physical symptoms, perhaps perceived as less stigmatising [23]. Socioeconomic factors could also play a role [3], as vulnerable populations may have less access to mental health services within the Swiss healthcare system due to language and financial reasons.

As expected, established CVRFs were less frequent in FSM patients [16]. In contrast, the history of psychiatric disorders was significantly more common in FSMs, consistent with previous findings [16]. Moreover, about half the FSM patients had a history of functional neurological and non‐neurological disorders, some of which had not been previously diagnosed. As this information was not collected for the AIS control group, the relative magnitude of this association cannot be estimated, but it appears to be substantial.

The overall clinical presentation of our FSM cohort was consistent with previous reports, except for decreased vigilance. Indeed, decreased vigilance, which included fluctuations of alertness and (pre‐)syncopal symptoms, emerged as a predictive factor of FSM in our population when compared with AIS patients; this has previously been observed in acute functional disorders [17] and should not be confused with the consciousness impairment that can be observed in severe AIS. Positive functional signs and clinical findings inconsistent with structural neurological lesions were recorded in all but one FSM patient during the emergency evaluation, which is a higher rate than previously reported [5, 13]. Importantly, the neurological presentation observed in FSM patients, often not corresponding to a vascular territory, represents an important clinical clue that has not yet been reported to the best of our knowledge.

Not surprisingly, acute multimodal CT did not reveal significant abnormalities in the large majority of FSM patients, a finding consistent with previous reports [16]. One FSM patient had slight right hemispheric benign oligaemia (Figure S2), a finding that remains unexplained. Indeed, the clinical presentation was fully consistent with a functional disorder; the patient had a prior history of multiple functional symptoms, and no features suggested an alternative or concomitant condition that could account for the perfusion abnormality, such as migrainous aura [24] or epileptic seizure [25].

Acute revascularisation treatment rates were similar between the two study groups but considerably higher for IVT in the FSM group than in most previously reported cohorts [3, 15, 16]. The relatively high rate of ‘over‐thrombolysis’ in our FSM patients may reflect a bias towards more stroke‐like presentations, as our inclusion criteria required that an acute PCT had been done, which clinicians are more likely to request when the clinical suspicion of stroke is high. Furthermore, the importance of minimising door‐to‐needle time for IVT [26] leaves limited time for detailed history‐taking or thorough assessment for functional signs.

Following our observations and those of previous studies, negative findings on perfusion CT and/or MRI‐based imaging in non‐lacunar presentations should raise the suspicion of stroke mimic, including FSM. We have previously shown that the use of advanced neuroimaging in acute stroke evaluation reduces the rate of over‐thrombolysis in stroke mimics by half [27]. Checking for a prior history of unexplained neurological symptoms or psychiatric comorbidity might also improve identification of these patients in the emergency setting. Greater emphasis should also be given to the evaluation of positive functional signs in neurology training so that they can be efficiently and confidently assessed, even in an emergency setting when a functional disorder is suspected.

The swift identification of FSM not only spares valuable resources but may also positively influence their long‐term outcome. Indeed, it has been suggested that a strong medical response to specific somatic diagnoses (including targeted investigations and treatments for presumed somatic disorders) may reinforce patients' attribution to physical illnesses, thereby perpetuating symptoms and contributing to the chronicity of the functional disorder [28]. The shorter median length of stay in our FSM patients compared to the control group could carry a risk of having less time to discuss FSM‐related issues during the acute hospital phase in the stroke unit. This, together with the high IVT rate, may put the patients at risk of not understanding that they suffer from a functional neurological disorder which could ultimately influence outcome. We therefore strongly suggest communicating in clear language and establishing specific pathways for in‐ and post‐hospital care of such patients.

Although the initial clinical course in FSM may suggest a favourable prognosis, long‐term follow‐up data in our cohort were less encouraging. Persistent symptoms—often perceived by patients as significant—and recurrent functional episodes were observed in more than half of the cases. Notably, most recurrences were again compatible with FSM. This data highlights the chronic nature of FND, of which FSM does not seem to be an exception. Based on our observations, FSM may be considered as the ‘tip of the functional iceberg’, reflecting a broader, long‐lasting condition in many patients. It is however worth considering that the long‐term handicap of our patients, recruited between 2003 and 2017, is possibly worse than it would be today, where a more proactive management of functional neurological disorders seems to improve such patients' prognosis [29, 30].

Limitations of this study include its small sample size and its retrospective, observational design. As a single‐centre quality assurance project conducted in a predominantly white population, the findings may not be applicable to more diverse settings and require confirmation in other populations. The requirement of an acute PCT and admission to the stroke unit as selection criteria may have introduced a selection bias towards more severe and complex presentations, and does not allow an estimation of prevalence of FSM in our stroke centre. Also, underestimation and misclassification of psychiatric comorbidities may have occurred given that 10/25 FSM patients did not undergo psychiatric evaluation during the acute hospital period. Moreover, long‐term outcome data were mainly patients' self‐reported information, which, while inherently subjective and less precise, remain valuable for capturing the patient's perceived burden and lived experience. Lastly, retrospective analysis of routine medical records may be less reliable than prospectively collected data.

In conclusion, in this single‐centre cohort of a limited number of FSM patients, we found that younger age and a history of psychosis and/or depressive disorders were more frequently observed among those ultimately diagnosed with functional symptoms rather than acute ischaemic stroke. IVT rates were high—though notably without bleeding complications—stressing the diagnostic challenge posed by FSM despite the frequent presence of positive functional signs and normal PCT findings. Long‐term follow‐up revealed a substantial rate of recurrent functional events and persistent disability. Future research should aim to develop a diagnostic framework incorporating demographics, medical history, clinical and imaging features, to aid early identification of FSM in the hyperacute setting and improve decision making and management.

## Author Contributions

**Selma Aybek:** writing – review and editing, validation, supervision. **Patrik Michel:** conceptualization, investigation, writing – original draft, methodology, validation, writing – review and editing, project administration, supervision. **Davide Strambo:** methodology, writing – review and editing, formal analysis, software, investigation. **Filipa Bastos:** conceptualization, investigation, writing – original draft, writing – review and editing, project administration, data curation, methodology, formal analysis. **Alexander Salerno:** investigation. **Vincent Dunet:** writing – review and editing, formal analysis, validation, methodology.

## Funding

There was no specific funding for this project; F.B. has no disclosures to report; D.S. has grant support from University of Lausanne and Swiss Heart Foundation; A.S. has no disclosures to report; V.D. has no disclosures to report; S.A. has no disclosures to report; P.M. reports within the last 2 years research grants from the Swiss National Science Foundation, the Swiss Heart Foundation, Faculty of Biology and Medicine of the University of Lausanne, and the Porphyrogenis Foundation.

## Conflicts of Interest

The authors declare no conflicts of interest.

## Supporting information


**Data S1:** Supplementary methods.

**Figure S1:** Flow diagram showing the reasons for exclusion of acute ischaemic stroke (AIS) patients from the control group because of non‐availability of a good quality perfusion computed tomography (PCT) within 24 h (ASTRAL = Acute STroke Registry and Analysis of Lausanne).

**Figure S2:** Computed tomography (CT) of the only FSM patient showing asymmetrical perfusion CT. (A) Plain CT, showing chronic lacunes in the right basal ganglia. (B) Perfusion CT, time‐to‐peak maps, showing slight slowing of perfusion in the right hemisphere (scale in seconds) corresponding to benign oligaemia. (C) Perfusion CT, cerebral blood flow, showing no significant abnormalities (scale in ml/100 g/min).

**Table S1:** Univariate analysis of clinical presentation. Continuous and ordinal variables are expressed as medians (with interquartile range, IQR), and categorical variables as absolute counts (with percentage), unless stated otherwise.

**Table S2:** Psychiatric and functional comorbidities of the functional stroke mimic cohort.

## Data Availability

The raw, anonymised data that support the findings of this study are available from the corresponding author upon reasonable request and after signing a data transfer and use agreement. If such data are used for a publication, its methods should be communicated, and internationally recognised authorship rules should be applied.
