# Supplementary material for: Functional Stroke Mimics: Patient Characteristics, CT‐Based Multimodal Imaging and Long‐Term Outcome in a Comparative Cohort Study
Source: Eur J Neurol. 2026 May 6;33(5):e70617. doi: 10.1111/ene.70617 (PMC13145337; doi:10.1111/ene.70617)
Supplement: Supplementary file 1 — Data S1: Supplementary methods. [file ENE-33-e70617-s003.docx]

**Supplementary material – Revision 1**

**For:** **Functional stroke mimics: patient characteristics, CT-based multimodal imaging and long-term outcome in a comparative cohort study**

Filipa Bastos, Davide Strambo, MD, Alexander Salerno, MD, PhD, Vincent Dunet, MD, Selma Aybek Rusca, MD, Patrik Michel, MD

**Supplementary methods**

Multimodal CT at CHUV for acute ischemic stroke

Multimodal CT-based imaging, including non-contrast CT (NCCT), CT-perfusion (CTP), CT-angiography (CTA) and post-contrast series, was performed in all patients with suspected AIS as part of standard of care, unless contrast contraindication existed. CTs were performed on a 16-detector CT scanner until November 2005, on a 64-multidetector CT scanner (LightSpeed VCT, GE Healthcare, Milwaukee, WI, USA) between November 2005 and 2015, or on a 256-multidetector CT scanner (Revolution CT, GE Healthcare, Milwaukee, WI, USA) thereafter.

*NCCT and post-contrast*: We acquired NCCT and post-contrast series in axial scan mode from the skull base to the vertex (16cm z-axis coverage) using the following imaging parameters: 120kV peak tube voltage, 320mA tube current, 5mm slice thickness, 32cm scan field of view (SFOV), 512x512 matrix.

*CTP:* All CTP series were acquired in axial scan mode with 80kV peak tube voltage, 240mA tube current, 32cm SFOV and 512x512 matrix. Images were positioned at the level of the basal ganglia and third ventricle above the orbits. We used 18 groups of 4 slices of 10mm (40mm z-axis coverage) until November 2005, 16 slices of 5mm (80mm z-axis coverage) from November 2005 to November 2015 and 24 slices of 5mm (120mm z-axis coverage) thereafter. CTP images were acquired for 50s in a cine mode before January 2011 and in a shuttle mode thereafter with a delay of 5-7 s from the beginning of injection of 50ml of iodinated contrast (Accupaque 300, iohexol 300mg/ml, GE Healthcare, Glattbrugg, Switzerland) in an antecubital vein at a flow rate of 5ml per second followed by 50ml of 0.9% NaCl solution at the same flow rate.

*CTA*: CTA was acquired in helical scan mode (120kV peak tube voltage, 150-260mA tube current, 0.984 pitch, 0.625mm slice thickness, 50cm SFOV, 512x512 matrix) from the aortic arch to the top of the frontal sinuses after injection of 50ml of iodinated contrast at a flow rate of 5ml/s followed by 50ml of 0.9%NaCl solution (time according to the perfusion data).

*CT reconstruction*: Raw data were reconstructed in the axial plane using filtered-back-projection (FBP) before February 2009 and using a blend of adaptive statistical iterative reconstruction (ASiR) and FBP thereafter (60%ASiR/40%FBP for NCCT and post-contrast series; 40%ASiR/60%FBP for CTP; 20%ASiR/80%FBP for CTA).

*CTP analysis*: CTP raw data were processed using the Brilliance Workspace Portal® (Philips Medical Systems, Cleveland, OH, USA) deconvolution software to create parametric maps of time to peak (TTP), mean transit time (MTT), cerebral blood flow (CBF) and cerebral blood volume (CBV). The CTP infarct core and ischemic penumbra volumes were automatically generated using the appropriate MTT and CBV thresholds (perfusion CT penumbra: MTT > 145% of the contralateral side value, CBV > 2.0 mL/100 g; perfusion CT infarct: MTT > 145% of the contralateral side value, CBV < 2.0 mL/100 g). Mismatch ratio (MR) was calculated as the ratio of the total ischemic volume (i.e. core plus penumbra volumes, i.e. hypoperfusion volume) to the core volume.

**Follow-up telephone interview Guide**

**A) General Information**

Patient Code:

Date of hospitalization at CHUV for the initial event:

Date of successful phone call:

Who is the person being interviewed?

□ Patient themselves

□ Other person(s) and relationship to the patient: _______________ If the patient is unable to answer the questions, is this person the legal representative of the patient? __

**B) Questions**

1. Has the patient passed away?

□ No

□ Yes. If yes:

Date of death

Cause/circumstances of death?

Place of death?

1. Authorisation: "You (or your relative) were hospitalized at CHUV in ... Would you (or you as the legal representative) agree that we use your data from that period, already archived at CHUV, for research purposes? Additionally, would you agree to answer a few questions for research purposes?"
2. What was the patient's disability following the stroke after hospital discharge (*Ranking scale evaluation guide provided to the interviewer*)?

Rankin Scale at 3 months:

Rankin Scale at 12 months:

Current Rankin Scale (at .....months):

1. Recurrence: Since leaving CHUV, have you (or your relative) experienced any other new acute neurological symptoms? (vision, speech, focal weakness, focal sensory disturbances, acute dizziness)

□ No

□ Yes. If yes:

Date(s): ________________ What symptom(s)? ___________________

1. Recurrence: Since leaving CHUV, have you (or your relative) received a diagnosis of:

□ Stroke (AIS), "warning" (TIA, attack lasting < 24 hours), transient monocular blindness (loss of vision in one eye), retinal infarction

□ Psychiatric/psychological problem

□ Other neurological disease (e.g., multiple sclerosis, epilepsy)

If yes, what and on what date(s)?

1. Recurrence: Since leaving CHUV, have you (or your relative) been hospitalised for any reason (i.e., spent at least one night in a hospital or emergency department), including in a psychiatric hospital?

□ No

□ Don't know

□ Yes, for stroke/TIA

□ Yes, for psychiatric/psychological problem

□ Yes, for other neurological disease

If yes, on what date(s)? ___________ Where (which hospital)? __________________ Why? (description): ______________________

1. After leaving CHUV, have you had any other significant health problems (including psychiatric)?

□ No

□ Yes: details? ____
