# Supplementary material for: Functional Stroke Mimics: Patient Characteristics, CT‐Based Multimodal Imaging and Long‐Term Outcome in a Comparative Cohort Study
Source: Eur J Neurol. 2026 May 6;33(5):e70617. doi: 10.1111/ene.70617 (PMC13145337; doi:10.1111/ene.70617)

**Tables and figures – Revision 1**

**For: Functional stroke mimics: patient characteristics, CT-based multimodal imaging and long-term outcome in a comparative cohort study**

Filipa Bastos, Davide Strambo, MD, Alexander Salerno, MD, PhD, Vincent Dunet, MD, Selma Aybek Rusca, MD, Patrik Michel, MD

**Supplementary figure 1S :** Flow diagram showing the reasons for exclusion of acute ischaemic stroke (AIS) patients from the control group because of non-availability of a good quality perfusion computed tomography (PCT) within 24 hours (ASTRAL = Acute STroke Registry and Analysis of Lausanne)


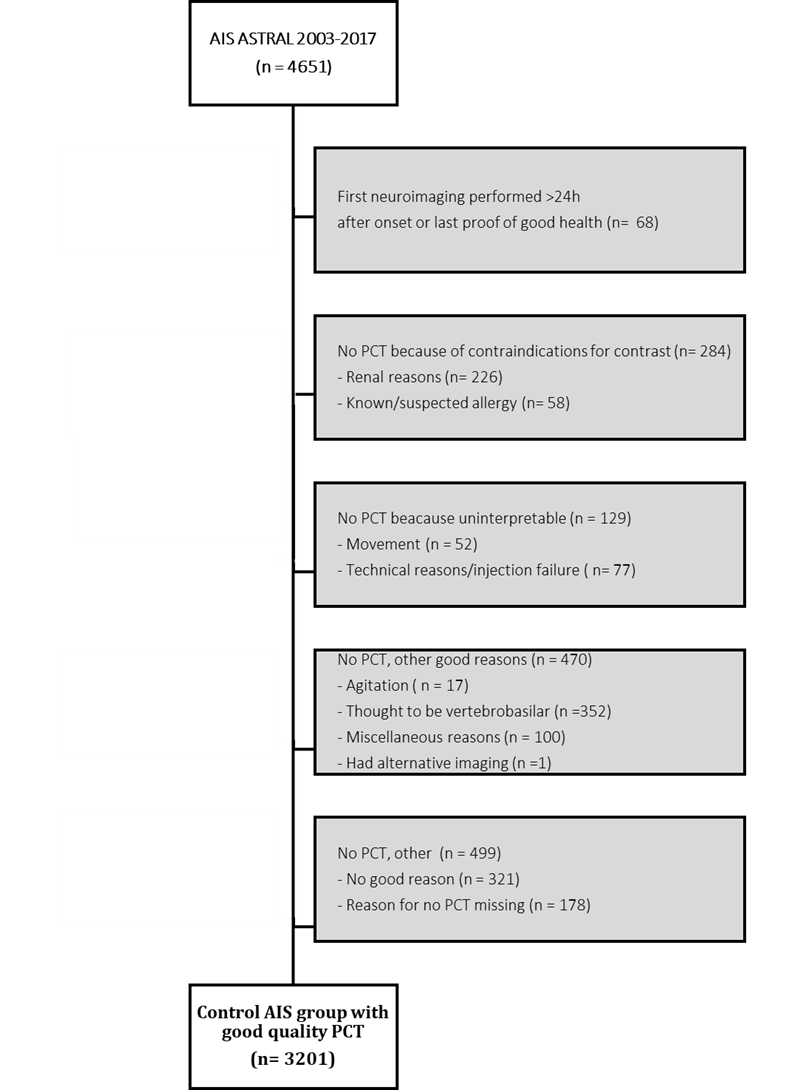

Supplement: Supplementary file 2 — Figure S1: Flow diagram showing the reasons for exclusion of acute ischaemic stroke (AIS) patients from the control group because of non‐availability of a good quality perfusion computed tomography (PCT) within 24 h (ASTRAL = Acute STroke Registry and Analysis of Lausanne). [file ENE-33-e70617-s001.docx]
