# Supplementary material for: Functional Stroke Mimics: Patient Characteristics, CT‐Based Multimodal Imaging and Long‐Term Outcome in a Comparative Cohort Study
Source: Eur J Neurol. 2026 May 6;33(5):e70617. doi: 10.1111/ene.70617 (PMC13145337; doi:10.1111/ene.70617)

**Tables and figures – Revision 1**

**For: Functional stroke mimics: patient characteristics, CT-based multimodal imaging and long-term outcome in a comparative cohort study**

Filipa Bastos, Davide Strambo, MD, Alexander Salerno, MD, PhD, Vincent Dunet, MD, Selma Aybek Rusca, MD, Patrik Michel, MD

**Supplementary figure 2S :** Computed tomography (CT ) of the only FSM patient showing asymmetrical perfusion CT. **A)** Plain CT, showing chronic lacunes in the right basal ganglia. **B)** Perfusion CT, time-to-peak maps, showing slight slowing of perfusion in the right hemisphere (scale in seconds) corresponding to benign oligaemia. **C)** Perfusion CT, cerebral blood flow, showing no significant abnormalities (scale in ml/100gm/min).


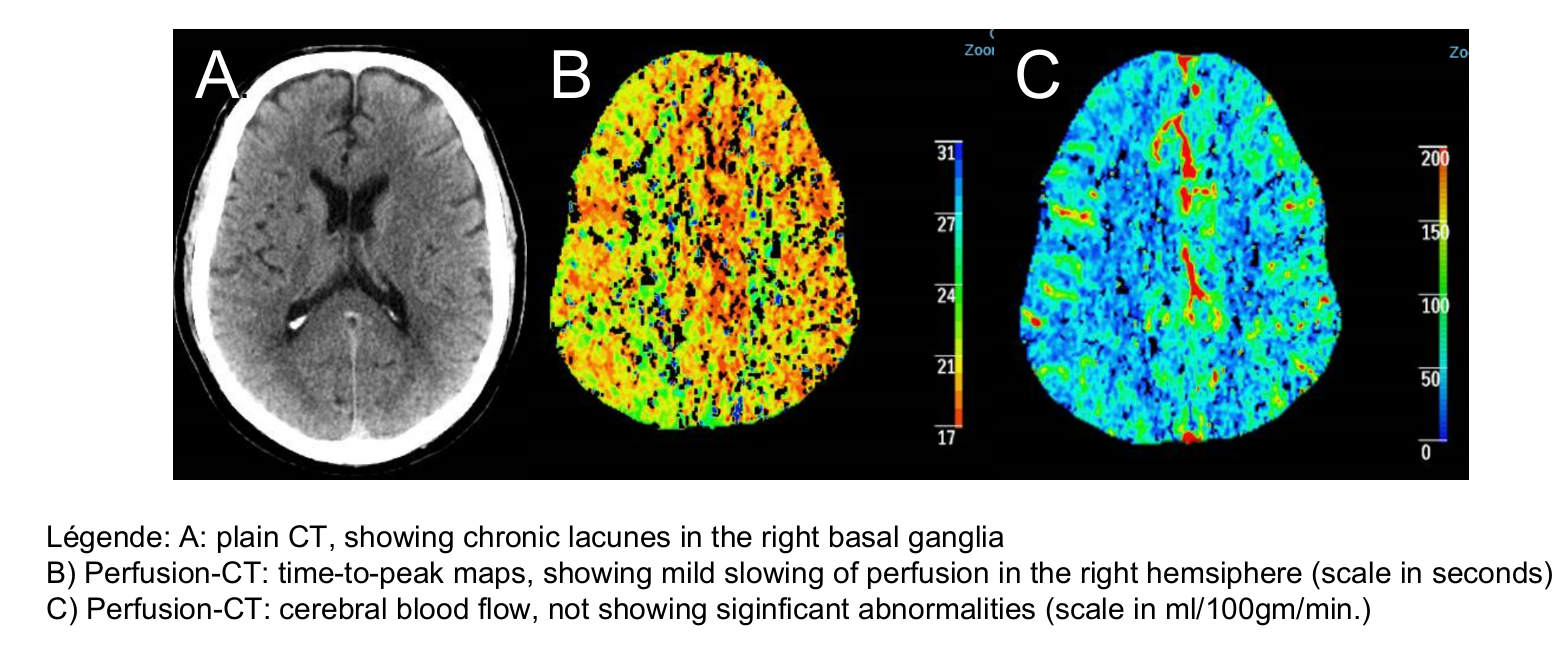

Supplement: Supplementary file 3 — Figure S2: Computed tomography (CT) of the only FSM patient showing asymmetrical perfusion CT. (A) Plain CT, showing chronic lacunes in the right basal ganglia. (B) Perfusion CT, time‐to‐peak maps, showing slight slowing of perfusion in the right hemisphere (scale in seconds) corresponding to benign oligaemia. (C) Perfusion CT, cerebral blood flow, showing no significant abnormalities (scale in ml/100 g/min). [file ENE-33-e70617-s004.docx]
