# Supplementary material for: Functional Stroke Mimics: Patient Characteristics, CT‐Based Multimodal Imaging and Long‐Term Outcome in a Comparative Cohort Study
Source: Eur J Neurol. 2026 May 6;33(5):e70617. doi: 10.1111/ene.70617 (PMC13145337; doi:10.1111/ene.70617)
Supplement: Supplementary file 4 — Table S1: Univariate analysis of clinical presentation. Continuous and ordinal variables are expressed as medians (with interquartile range, IQR), and categorical variables as absolute counts (with percentage), unless stated otherwise. [file ENE-33-e70617-s005.docx]

**Tables and figures – Revision 1**

**For: Functional stroke mimics: patient characteristics, CT-based multimodal imaging and long-term outcome in a comparative cohort study**

Filipa Bastos, Davide Strambo, MD, Alexander Salerno, MD, PhD, Vincent Dunet, MD, Selma Aybek Rusca, MD, Patrik Michel, MD

**Supplementary table 1:** Univariate analysis of clinical presentation. Continuous and ordinal variables are expressed as medians (with interquartile range, IQR), and categorical variables as absolute counts (with percentage), unless stated otherwise.

| **Variable** | **Overall population**  **(n=3226)** | **AIS**  **(n=3201)** | **FSM**  **(n=25)** | **P-value**  **(univ)** | **Crude odds ratio (95%CI)** |
| --- | --- | --- | --- | --- | --- |
| Clinical presentation |  |  |  |  |  |
| Vigilance alteration | 394/3226 (12.2%) | 386/3201 (12.1%) | 8/25 (32%) | 0.01 | 3.43 (1.47-8.01)* |
| Visual field defect | 1322/3210 (41.2%) | 1318/3185 (41.4%) | 4/25 (16%) | 0.02 | 0.27 (0.09-0.79)* |
| Eye deviation | 917/3215 (28.5%) | 913/3190 (28.6%) | 4/25 (16%) | 0.24 | 0.48 (0.16-1.39) |
| Brainstem oculomotor defect | 259/3220 (8%) | 259/3195 (8.1%) | 0/25 (0%) | 0.26 | NA |
| Facial or limb paresis | 2687/3223 (83.4%) | 2665/3198 (83.3%) | 22/25 (88%) | 0.72 | 1.47 (0.44-4.92) |
| Sensory deficit | 1730/3203 (54%) | 1712/3178 (53.9%) | 18/25 (72%) | 0.11 | 2.20 (0.92-5.29) |
| Cerebellar deficit | 799/3195 (25%) | 794/3170 (25%) | 5/25 (20%) | 0.73 | 0.75 (0.28-2.00) |
| Dysarthria | 1822/3220 (56.6%) | 1816/3195 (56.8%) | 6/25 (24%) | <0.01 | 0.24 (0.10-0.60)* |
| Aphasia | 1267/3222 (39.3%) | 1260/3197 (39.4%) | 7/25 (28%) | 0.34 | 0.60 (0.25-1.44) |
| Hemi-neglect | 859/3207 (26.8%) | 856/3182 (26.9%) | 3/25 (12%) | 0.15 | 0.37 (0.11-1.24) |
| Other cognitive deficit | 371/3211 (11.6%) | 368/3186 (11.6%) | 3/25 (12%) | 1 | 1.04 (0.31-3.51) |
| Functional signs/symptoms |  |  |  |  |  |
| According to neurologist’s judgment | 24/25 (96%) | NA | 24/25 (96%) | NA | NA |
| According to Popkirov *et al* 2020, Stroke [7] | 10/25 (40%) | NA | 10/25 (40%) | NA | NA |
| Arterial territory ‡ |  |  |  | <0.01 |  |
| Anterior circulation | 2388/3226 (74%) | 2381/3201 (74.4%) | 7/25 (28%) | <0.01 | Ref. |
| Posterior circulation | 639/3226 (19.8%) | 631/3201 (19.7%) | 8/25 (32%) | 0.2 | 4.31 (1.56-11.94) |
| Ant + Post circulation | 61/3226 (1.9%) | 61/3201 (1.9%) | 0/25 (0%) | 1 | 0.00 (0.00-Inf) |
| Undetermined | 138/3226 (4.3%) | 128/3201 (4%) | 10/25 (40%) | <0.01 | 26.57 (9.95-70.95) |

‡ Arterial territory determined according to clinical findings in FSM and according to clinical and positive radiological findings in AIS control group. AIS = Acute ischaemic stroke; FSM = Functional stroke mimic; NA = not available or not applicable; Ref. = reference for odds ratio calculation
