# Supplementary material for: Functional Stroke Mimics: Patient Characteristics, CT‐Based Multimodal Imaging and Long‐Term Outcome in a Comparative Cohort Study
Source: Eur J Neurol. 2026 May 6;33(5):e70617. doi: 10.1111/ene.70617 (PMC13145337; doi:10.1111/ene.70617)
Supplement: Supplementary file 5 — Table S2: Psychiatric and functional comorbidities of the functional stroke mimic cohort. [file ENE-33-e70617-s002.docx]

**Tables and figures – Revision 1**

**For: Functional stroke mimics: patient characteristics, CT-based multimodal imaging and long-term outcome in a comparative cohort study**

Filipa Bastos, Davide Strambo, MD, Alexander Salerno, MD, PhD, Vincent Dunet, MD, Selma Aybek Rusca, MD, Patrik Michel, MD

**Supplementary table 2 :** Psychiatric and functional comorbidities of the functional stroke mimic cohort.

| Study ID | History of psychiatric disorders  (*Elixhauser code*) | In-hospital psychiatric evaluation  (*conclusion*) | History of functional disorders  (*details*) | Long-term functional disorders recurrences |
| --- | --- | --- | --- | --- |
| 10001 | No | No | No | No data |
| 10002 | No | Yes  (*Histrionic personality traits*) | No | Chronic fatigue  Several episodes of paraesthesia and/or paresis (including “locked-in”-like syndrome) |
| 10003 | No | Yes  (*Probable somatoform disorder*) | No | Chronic headaches  Several episodes of vertigo and/or paraesthesia and/or hemiparesis |
| 10004 | No | Yes  (*Has ability to discern*) | No | No data |
| 10005 | No | No | No | No recurrences |
| 10006 | Depressive disorder (*29*) | Yes  (*Conversion disorder*  *Post traumatic stress disorder following childhood sexual abuse with emotional regulation difficulties and avoidance behaviour*) | Yes  (E*pisode of blindness and loss of conscience*) | Chronic headaches  Several episodes of loss of vision or blurred vision |
| 10007 | Depressive disorder (*29*)  Borderline personality disorder (*30*)  Gender identity disorder | Yes  (*Probable functional disorder Alcohol dependence syndrome, currently abstinent in protected environment*) | Yes  (*Chronic pain in right inferior limb*  *Functional gait disorder*) | No recurrences |
| 10008 | No | No | No | No recurrences |
| 10010 | Recurrent mood disorder (*29*) | Yes  (*Recurrent depressive disorder, current episode mild*  *Motor dissociative disorder*) | Yes  (*Multi-investigated syncopal episodes*) | Several episodes of aphasia + left hemisyndrome triggered by stress or alcohol |
| 10011 | Mixed anxiety and depressive disorder with panic attacks (*29*) | No | Yes  (*Self-resolving episode of left upper limb paresis, imaging negative*) | No recurrences |
| 10012 | Episodic depressive disorder under pharmacological treatment (*29*) | No | No | No recurrences |
| 10013 | No | No | Yes  (*Episode of left hemi-paresis and hemi-paraesthesia*) | No data |
| 10014 | No | No | Yes  (PNES  Previous self-resolving episode of right motor and sensitive hemisyndrome + aphasia after conflict with husband  Fibromyalgia) | Functional stroke-like episode in 2012  Several episodes of PNES |
| 10016 | Emotional lability (30) | No | Yes  (*Several self-resolving episodes of motor and sensitive hemisyndromes*  *Recurrent episodes of thoraix pain, multi-investigated including explorative sternotomy, with no cause found*) | Episode of aphasia + right hemisyndrome in 2016 |
| 10017 | No | No | Yes  (*Self-resolving Tetanic episode with tetraplegia during university exams*) | No recurrences |
| 10018 | Borderline personality disorder (*30*) | Yes  (*Personality disorder, unspecified Severe depressive episode Withdrawal syndrome*) | No | No recurrences |
| 10019 | No | Yes  (*Probable functional disorder*) | Yes  (*FSM in 2012 with acute admission and rehabilitation)* | Self-resolving functional left hemisyndrome triggered by dispute in 2014  Self-resolving Episode of retroorbital pain with loss of sensibility in left hemi-face in 2020 |
| 10020 | No | Yes  (Problems in relationship with spouse or partner  Suffering related with several factors (retirement, child accident, conflict with spouse)) | No | No recurrences |
| 10021 | No | No evaluation | Yes  (*Episode of self-resolving mutism after aggression*) | No recurrences |
| 10022 | No | Yes  (*Dissociative disorder*) | No | At least 3 episodes of headache + blurred vision + right hemisyndrome +/- amnesia/confusion +/- aphasia of functional origin (with hospitalisation) |
| 10023 | Depressive disorder 20 year before event (*29*)  Anxiety disorder with acute over-breathing episodes (*30*) | Yes  (*Mixed dissociative disorder*) | Yes  (*Self-resolving left side motor hemisyndrome in 2012*) | No recurrences |
| 10024 | Anxiety and depressive symptoms  (*30*) | Yes  (*Recurrent depressive disorder, current episode moderate with somatic symptoms*  *Somatization disorder*) | Yes  (*Functional thoracic pain episodes*) | Chronic vertigo and fatigue |
| 10025 | Mixed anxiety and depressive disorder (*29*) | Yes  (*Mixed anxiety and depressive disorder*) | No | Several episodes of functional stroke-like episodes |
| 10026 | Professional stress, burnout (*30*) | Yes  (*Mixed (motor and sensitive) dissociative disorder*) | No | Several self-resolving episodes of “blockage” of right side of body triggered by stress |
| 10028 | Mixed anxiety and depressive disorder (*29*)  Personality disorder (*30*)  Scarifications | Yes  (*Probable conversion disorder*  *Recurrent depressive disorder, current episode moderate*  *Personality disorder, unspecified*) | Yes  (Several episodes of “somatisation”) | Functional stroke-like episode in 2019 |

PNES = psychogenic non epileptic seizures
